# Trends and variation in the use of andexanet alfa for the reversal of direct oral anticoagulants in NHS trusts in England

**DOI:** 10.1002/bcp.70417

**Published:** 2025-12-17

**Authors:** Louis Fisher, Richard Buka, Rosalind Byrne, Stephen Black, Helen J. Curtis, Christopher Wood, Andrew Brown, Sebastian Bacon, Richard Croker, Ben Goldacre, Brian MacKenna, Victoria Speed

**Affiliations:** ^1^ Bennett Institute for Applied Data Science, Nuffield Department of Primary Care Health Sciences University of Oxford Oxford UK; ^2^ Department of Cardiovascular Sciences University of Birmingham Birmingham UK; ^3^ Haematology, King's Thrombosis Centre King's College Hospital NHS Foundation Trust London UK

**Keywords:** anticoagulant reversal agents, anticoagulants, antidotes, thrombosis

## Abstract

**Aims:**

Andexanet alfa was recommended by the National Institute for Health and Clinical Excellence (NICE) as an option in the management of life‐threatening gastrointestinal bleeding in patients taking apixaban or rivaroxaban in May 2021. A recent UK‐wide survey of local hospital protocols for the use of andexanet alfa suggested that practice across the United Kingdom is highly variable. In January 2025, NICE was unable to make a recommendation on the use of andexanet alfa for reversing anticoagulation in adults with intracranial haemorrhage. We set out to report trends and variation in the uptake of andexanet alfa across the NHS in England, using the new OpenPrescribing Hospitals platform.

**Methods:**

To assess the uptake of andexanet alfa, we analysed pharmacy stock control data from NHS trusts in England using the openly available Secondary Care Medicines Dataset. Analysis was restricted to NHS trusts with 24‐h consultant‐led emergency care activity.

**Results:**

Between May 2021 and June 2025, 19 608 vials of andexanet alfa were issued in NHS trusts in England. There was wide variation in the timing and speed of uptake across NHS trusts. Substantial variation was also observed between NHS Regions in England.

**Conclusions:**

The use of andexanet alfa varies markedly between NHS regions and between NHS trusts in England. This study is the first analysis of adoption of a new treatment using the OpenPrescribing Hospitals platform, which is expected to provide a generalizable framework for similar analyses in the future.

**What is already known about this subject?**

- Andexanet alfa was approved by NICE for the reversal of apixaban and rivaroxaban in life‐threatening gastrointestinal bleeding in 2021.
- In 2025, NICE announced that it was unable to make a recommendation about the use of andexanet alfa for reversing anticoagulation in adults with intracranial haemorrhage in the NHS.
- Andexanet alfa is expensive, and a survey of local hospital trust protocols suggested that uptake would be mixed.

**What this study adds?**

- This study shows substantial variation in the speed and extent of uptake of andexanet alfa across hospital trusts in England.
- This study shows marked regional variation in the use of andexanet alfa.

## 1 INTRODUCTION

The National Institute for Health and Clinical Excellence (NICE) recommends andexanet alfa (AA) as an option in the management of life‐threatening gastrointestinal (GI) bleeding in patients taking apixaban or rivaroxaban (NICE TA697, May 2021).^1^ The recommendation was based on findings from the single‐arm ANNEXA‐4 trial where 90 patients with major GI bleeding received AA and 85% were found to have good or excellent haemostatic efficacy.^2^

A subsequent trial, ANNEXA‐I, randomized patients with DOAC‐associated intracranial haemorrhage to AA or usual care and found a reduction in haematoma expansion but no improvement in clinical outcomes including death or disability^3^; however, the trial was not powered for these outcomes. Both trials show that AA increases the risk of thrombosis, predominantly arterial. In January 2025, NICE confirmed it was not able to provide a recommendation about the use of AA in the NHS for reversing anticoagulation from apixaban or rivaroxaban in adults with intracranial haemorrhage.^4^

Andexanet alfa is also expensive.^5^ The National Health Service (NHS) list price is approximately £15 000 per patient. Whilst the NHS receives a discount for AA, the amount is commercially sensitive and confidential.^1^ In this context, a recent UK‐wide survey of local hospital protocols for the use of AA suggested that practice across the United Kingdom is highly variable.^6^

Although primary care prescribing data has been extensively used for variation in care analysis, until recently, there was no open access to hospital medicines usage data. As of July 2021, the NHS Business Services Authority has published the Secondary Care Medicines Data (SCMD), which contains data on the quantity of individual medicines and devices issued by all NHS trusts in England.^7^ This dataset requires domain and technical knowledge to access, process and link to other data sources before it can be analysed. We have developed the OpenPrescribing Hospitals (https://hospitals.openprescribing.net/) platform to remove these technical barriers and provide an easy‐to‐use interface to this data.^8^ The platform is free to use and available for use by any interested user.

To assess the adoption of this new high cost treatment by NHS hospitals and to demonstrate the potential of OpenPrescribing Hospitals, we set out to use the platform to report the volume of AA issued in secondary care trusts in England and identify regional trends and variation.

## 2 METHODS

We accessed AA usage data between May 2021 and June 2025 through the OpenPrescribing Hospitals platform.^9^, ^10^ The primary underlying data source is the Secondary Care Medicines Data (SCMD), collated by Rx‐info and published by the NHS Business Services Authority (NHSBSA).^7^ The SCMD contains issued pharmacy stock control data from all NHS Acute, Teaching, Specialist, Mental Health and Community trusts in England. The OpenPrescribing Hospitals Platform additionally links to the dictionary of medicines and devices (dm + d) and Organisation Data Service (ODS), which provide more detailed product and organization information.^11^, ^12^

Using publicly available A&E Attendances and Emergency Admissions data published by NHS England, we identified NHS trusts with 24‐h consultant‐led emergency care activity reported in the last 6 months (January 2025–June 2025).^13^ Trusts known to specialize in paediatric or women's health were excluded. Further analysis of AA usage was restricted to these trusts.

To assess regional variation in AA issuing, we calculated a 6‐month rolling average of the monthly quantity of issued vials broken down by NHS region. To investigate the issuing of AA within each NHS region relative to apixaban and rivaroxaban use, we extracted both primary care prescribing data from OpenPrescribing and secondary care issuing data from OpenPrescribing Hospitals for apixaban and rivaroxaban between May 2021 and June 2025.^14^, ^15^ For both, the quantity of apixaban and rivaroxaban prescribed or issued was converted to a quantity in Defined Daily Doses (DDDs) for all preparations where this is possible, using DDD values specified in the World Health Organization ATC/DDD index.^16^ A full list of the products captured can be found in Table S1. This was aggregated by NHS region.

For each NHS region, we then calculated the monthly number of AA vials issued by NHS trusts, standardized per 100 000 DDDs of apixaban and rivaroxaban, using combined primary and secondary care usage data. Both the absolute and relative number of AA vials issued are plotted as time series charts.

To identify trust‐level variation, we calculated percentiles each month using individual trust quantity values. Decile bands are plotted on a time series chart, on which a selection of individual trusts are overlaid.

The recommended dose of AA is five vials for the *low dose* regimen or nine vials for the *high dose* regimen.^17^ This was used to calculate the number of low and high dose regimens patients had been treated with across the study period.

All data are openly available.^7^ No ethical approval was sought for this analysis. Code for data management and analysis are openly available for inspection and re‐use at github.com/ebmdatalab/op‐hospitals‐andexanet.

### 2.1 Nomenclature of targets and ligands

Key ligands in this article are hyperlinked to corresponding entries in http://www.guidetopharmacology.org and are permanently archived in the Concise Guide to PHARMACOLOGY 2021/22.^18^

## 3 RESULTS

We identified 121 trusts with emergency care activity. Three trusts with paediatric or women's only emergency care activity were excluded (Table S2). A total of 19 608 vials of AA were issued between May 2021 and June 2025, with 117/118 (99.2%) eligible trusts issuing AA in the study period (Table S2). Assuming all vials had been administered, between 2179 (19 608/9 vials, high dose regimen) and 3921 (19 608/5 vials, low dose regimen), patients have been treated as of June 2025 in England.

**FIGURE 1:**
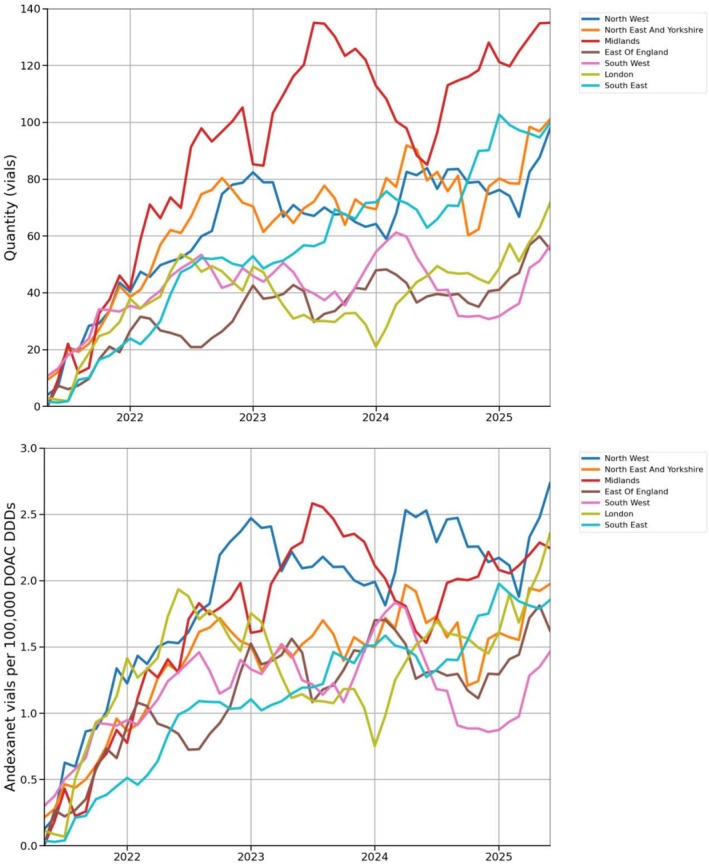
(A) The monthly number of vials of AA issued in secondary care between May 2021 and June 2025 by NHS region (6‐month rolling average). (B) The monthly number of vials of AA issued in secondary care per 100 000 DDDs of apixaban and rivaroxaban issued in both primary and secondary care, by NHS region (6‐month rolling average).

Figure 1A shows the 6‐month rolling average number of vials of AA issued in each of the seven NHS regions in England. There is regional variation in the total quantity of AA issued and the speed of uptake. The extent of variation has increased over time. From May 2022, most AA was issued in the Midlands. The number of vials of AA issued in each NHS region relative to the number of DDDs of apixaban and rivaroxaban issued in both primary and secondary care is shown in Figure 1B. The regional rate of AA issued relative to apixaban and rivaroxaban issuing is low, reaching a maximum monthly value of 2.7 vials/100 000 DDDs in the North West in June 2025. The relative issuing of AA shows similar regional variation, with the highest relative rates in the Midlands and the North West.

**FIGURE 2:**
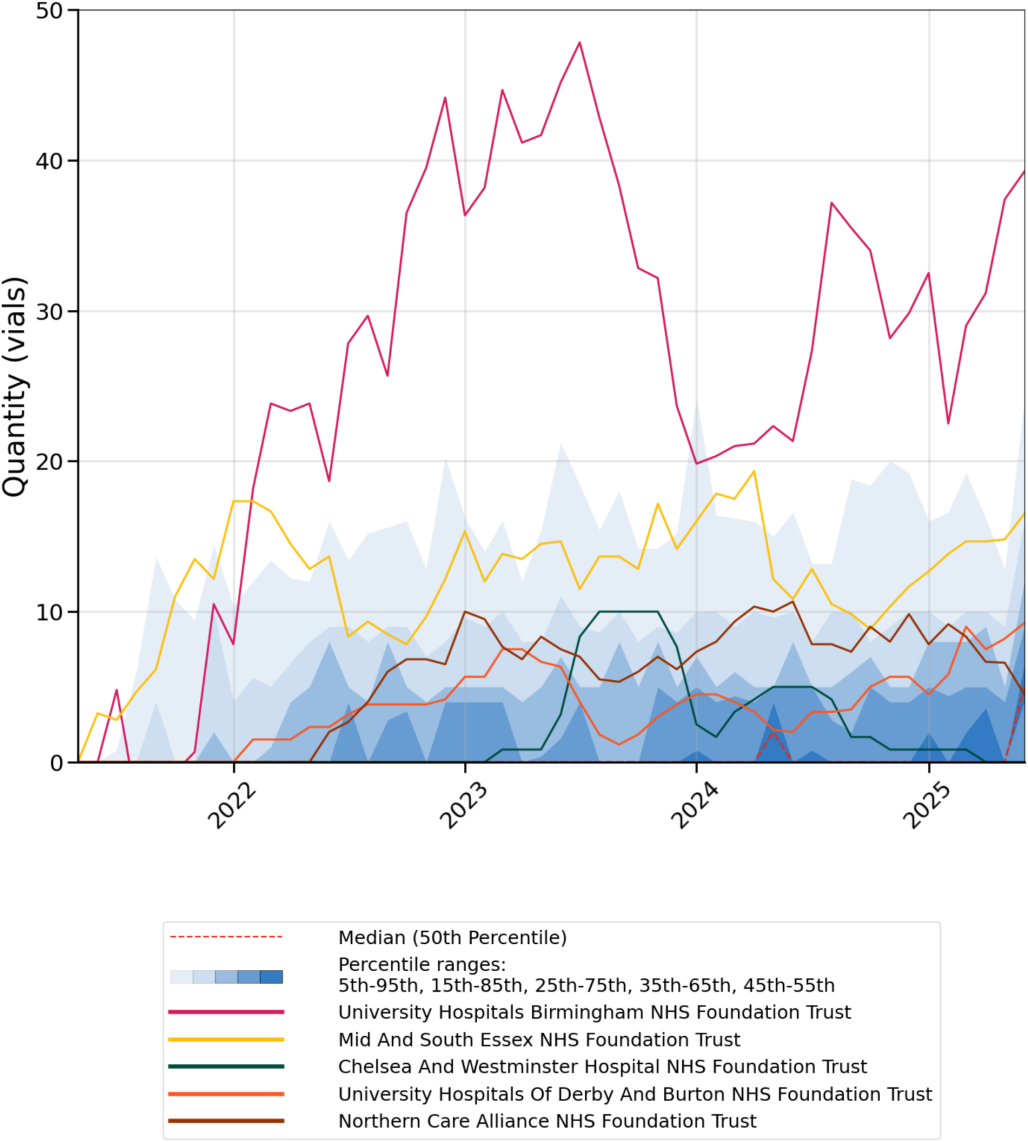
The monthly number of vials of AA issued in secondary care in five large NHS trusts between May 2021 and June 2025. The shaded deciles represent variation in monthly counts of AA vials issued across all included trusts. The individual trusts are reported as a 6‐month rolling average of the monthly counts of AA vials issued.

There was also variation in uptake amongst NHS trusts (Figure 2, note for interpretation of decile ranges that the trust median is zero until mid‐2024). Total usage since May 2021 for the 25 trusts with most usage is reported in Figure S1.

## 4 DISCUSSION

This is the first study to describe the marked variation in adoption of AA in England. Our data demonstrate widespread uptake of AA since the publication of the NICE TA in May 2021 and are consistent with AA being present on the Royal College of Emergency Physicians Antidote list^19^ but also suggest widespread low usage in many trusts. It is the first publication from the OpenPrescribing Hospitals platform,^9^ a new, openly accessible project to enable anyone to analyse medicines issued in NHS trusts England using open data; it builds on the successful delivery of openprescribing.net, an analytic platform for prescribing data in primary care with 20 000 users per month.^14^

The openly available SCMD is a rich data source. However, there are limitations in the use of stock control data where patient level medicines usage is not available. For this reason, we cannot determine whether the AA has been used for indications outside NICE recommendations, for example, intracranial haemorrhage. Where a trust has not issued AA, we cannot be sure whether this because it has not been issued yet (e.g., not been prescribed for a patient) or whether the trust does not hold stock. Similarly, we were unable to differentiate when AA was issued but not administered to a patient. Further, there are limited options for a suitable denominator when assessing medicines issued in secondary care. It is, for example, challenging to define the catchment population for each trust. Other trust‐level features, such as whether they are referral sites, are not available. However, the regional variation in AA issuing relative to apixaban and rivaroxaban use indicates that differences in DOAC prescribing alone do not fully account for the observed variation in AA use. Finally, actual cost is not reported in the SCMD (only indicative cost, which does not reflect the discounts applied by the manufacturer for the NHS).

The study highlights considerable variation in the uptake of a new, expensive agent. This wide variation is likely due to variation in local protocols, which are influenced by individual clinicians' interpretation of the evidence.^6^ This raises important questions for policy makers and those responsible for local clinical guidance implementation. Following the recent termination of the NICE technology appraisal for AA for DOAC‐associated intracranial haemorrhage, our tool provides an opportunity to repeat this analysis to measure the impact of the decision in hospital trusts in England in a future study. For clarity on the risk and benefit of AA for GI haemorrhage, a randomized controlled trial is needed: Until a trial is commissioned, the natural variation identified here may provide an opportunity to estimate effectiveness, using treating hospital as an instrumental variable. This study provides the first window into the adoption of a new high cost treatment by NHS hospitals, from a platform that is expected to produce a generalized service describing varied adoption of many such treatments in the future. Further research is required to understand the reasons why AA is used heavily in some centres and not at all in others.

## AUTHOR CONTRIBUTIONS

*Conceptualization*: Louis Fisher and Victoria Speed. *Data curation*: Louis Fisher and Stephen Black. *Formal analysis*: Louis Fisher and Victoria Speed. *Funding acquisition*: Ben Goldacre and Brian MacKenna. *Investigation*: Louis Fisher, Stephen Black, and Victoria Speed. *Methodology*: Louis Fisher, Stephen Black, Richard Croker, Ben Goldacre, and Brian MacKenna. *Project administration*: Victoria Speed. *Resources*: Sebastian Bacon. *Software*: Louis Fisher and Sebastian Bacon. Supervision: Ben Goldacre. *Validation*: Louis Fisher, Christopher Wood, Andrew Brown, Richard Croker, and Victoria Speed. *Visualization*: Louis Fisher, Ben Goldacre, and Victoria Speed. *Writing—original draft*: Louis Fisher, Richard Buka, Rosalind Byrne, and Victoria Speed. *Writing—review and editing*: Louis Fisher, Richard Buka, Rosalind Byrne, Stephen Black, Helen J. Curtis, Christopher Wood, Andrew Brown, Sebastian Bacon, Richard Croker, Ben Goldacre, Brian MacKenna, and Victoria Speed.

## CONFLICT OF INTEREST STATEMENT

All authors have completed the ICMJE uniform disclosure form at www.icmje.org/coi_disclosure.pdf and declare the following: BG has received research funding from the Laura and John Arnold Foundation, the NHS National Institute for Health Research (NIHR), the NIHR School of Primary Care Research, the NIHR Oxford Biomedical Research Centre, the Mohn‐Westlake Foundation, NIHR Applied Research Collaboration Oxford and Thames Valley, Wellcome Trust, the Good Thinking Foundation, Health Data Research UK, the Health Foundation, the World Health Organization, UKRI, Asthma UK, the British Lung Foundation and the Longitudinal Health and Wellbeing strand of the National Core Studies programme; he also receives personal income from speaking and writing for lay audiences on the misuse of science. BMK, RC, VS, CW and AB work for the NHS and are seconded to the Bennett Institute. The following authors are employed on BG's grants: LF, HJC, CW, AB, SB, RC, BMK and VS. VS has received speaker fees from Bayer. R Buka is a named investigator on an externally sponsored research grant from AstraZeneca to audit real‐world use of reversal agents for DOACs across the United Kingdom.

## Supporting information


**Figure S1.** **Top 25 NHS Trusts by number of vials of andexanet alfa used between May 2021 and June 2025.**
**Table S1.** Apixaban and rivaroxaban products available for analysis, with an indication of whether they are included in the analysis of primary care and secondary care data.
**Table S2.** NHS Trusts in the SCMD, with an indication of whether they had any emergency department activity in the last 6 months (and were therefore included in the analysis) and an indication of whether they issued any andexanet alfa between May 2021 and June 2025.

## Data Availability

These data were derived from the following resources available in the public domain: The Secondary Care Medicines Dataset, available at https://opendata.nhsbsa.net/dataset/finalised-secondary-care-medicines-data-scmd-with-indicative-price. NHS A&E Attendances and Emergency Admissions, available at: https://www.england.nhs.uk/statistics/statistical-work-areas/ae-waiting-times-and-activity/. NHS Organisation Data, available at https://www.odsdatasearchandexport.nhs.uk/. NHS dictionary of medicines and devices (dm + d), available at https://www.nhsbsa.nhs.uk/pharmacies-gp-practices-and-appliance-contractors/nhs-dictionary-medicines-and-devices-dmd. All code for the OpenPrescribing Hospitals platform, available at https://github.com/bennettoxford/openprescribing-hospitals. All code for this study, available at: https://github.com/ebmdatalab/op-hospitals-andexanet.
