# Supplementary material for: Trends and variation in the use of andexanet alfa for the reversal of direct oral anticoagulants in NHS trusts in England
Source: Br J Clin Pharmacol. 2025 Dec 17;92(6):1607–13. doi: 10.1002/bcp.70417 (PMC13206202; doi:10.1002/bcp.70417)
Supplement: Supplementary file 1 — Figure S1. Top 25 NHS Trusts by number of vials of andexanet alfa used between May 2021 and June 2025. Table S1. Apixaban and rivaroxaban products available for analysis, with an indication of whether they are included in the analysis of primary care and secondary care data. Table S2. NHS Trusts in the SCMD, with an indication of whether they had any emergency department activity in the last 6 months (and were therefore included in the analysis) and an indication of whether they issued any andexanet alfa between May 2021 and June 2025. [file BCP-92-1607-s001.docx]

## Supplementary material

**Figure S1. Top 25 NHS Trusts by number of vials of andexanet alfa used between May 2021 and June 2025**


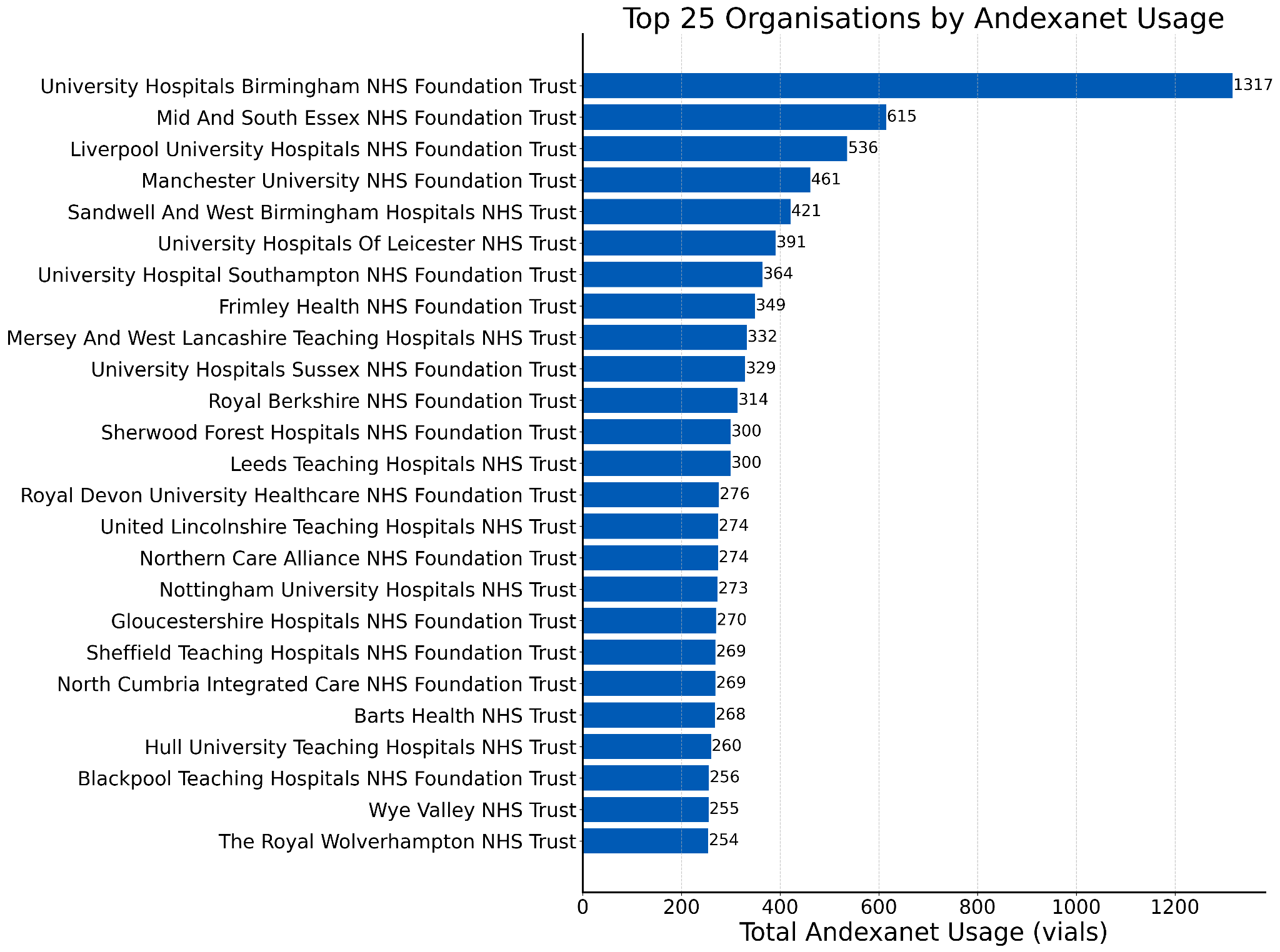


**Supplementary Table 1.** Apixaban and rivaroxaban products available for analysis, with an indication of whether they are included in the analysis of primary care and secondary care data.

| **Product name** | **SNOMED CT Code** | **Included in primary care data** | **Included in secondary care data** | **Notes** |
| --- | --- | --- | --- | --- |
| Rivaroxaban 15mg tablets and Rivaroxaban 20mg tablets | 34819111000001102 | N | N | Can’t calculate DDDs for combination product |
| Apixaban 2.5mg tablets | 42206411000001101 | Y | Y |  |
| Apixaban 5mg tablets | 42206511000001102 | Y | Y |  |
| Rivaroxaban 10mg tablets | 14254711000001104 | Y | Y |  |
| Rivaroxaban 2.5mg tablets | 27810711000001104 | Y | Y |  |
| Rivaroxaban 15mg tablets | 19842111000001101 | Y | Y |  |
| Rivaroxaban 20mg tablets | 19842211000001107 | Y | Y |  |
| Rivaroxaban 1mg/ml granules for oral suspension sugar free | 39609211000001101 | Y | Y |  |
| Rivaroxaban 10mg capsules | 42957511000001105 | Y | Y |  |
| Rivaroxaban 15mg capsules | 42957611000001109 | Y | Y |  |
| Rivaroxaban 20mg capsules | 42957711000001100 | Y | Y |  |
| Rivaroxaban 5mg/ml oral suspension sugar | 45160111000001108 | Y | N | Not issued in secondary care  (new product available since 21-05-02025) |

**Supplementary Table 2.** NHS Trusts in the SCMD, with an indication of whether they had any emergency department activity in the last 6 months (and were therefore included in the analysis) and an indication of whether they issued any andexanet alfa between May 2021 and June 2025.

| **ODS code** | **Trust name** | **Emergency department activity in the last 6 months*** | **AA issued** |
| --- | --- | --- | --- |
| RCF | Airedale NHS Foundation Trust | Yes | Yes |
| RBS | Alder Hey Children's NHS Foundation Trust | Yes (Paediatric   - not included) | No |
| RTK | Ashford And St Peter's Hospitals NHS Foundation Trust | Yes | Yes |
| RF4 | Barking, Havering And Redbridge University Hospitals NHS Trust | Yes | Yes |
| RFF | Barnsley Hospital NHS Foundation Trust | Yes | Yes |
| R1H | Barts Health NHS Trust | Yes | Yes |
| RC9 | Bedfordshire Hospitals NHS Foundation Trust | Yes | Yes |
| RQ3 | Birmingham Women's And Children's NHS Foundation Trust | Yes (Paediatric and women’s   - not included) | No |
| RXL | Blackpool Teaching Hospitals NHS Foundation Trust | Yes | Yes |
| RMC | Bolton NHS Foundation Trust | Yes | Yes |
| RAE | Bradford Teaching Hospitals NHS Foundation Trust | Yes | Yes |
| RXQ | Buckinghamshire Healthcare NHS Trust | Yes | Yes |
| RWY | Calderdale And Huddersfield NHS Foundation Trust | Yes | Yes |
| RGT | Cambridge University Hospitals NHS Foundation Trust | Yes | Yes |
| RQM | Chelsea And Westminster Hospital NHS Foundation Trust | Yes | Yes |
| RFS | Chesterfield Royal Hospital NHS Foundation Trust | Yes | Yes |
| RJR | Countess Of Chester Hospital NHS Foundation Trust | Yes | Yes |
| RXP | County Durham And Darlington NHS Foundation Trust | Yes | Yes |
| RJ6 | Croydon Health Services NHS Trust | Yes | Yes |
| RN7 | Dartford And Gravesham NHS Trust | Yes | Yes |
| RP5 | Doncaster And Bassetlaw Teaching Hospitals NHS Foundation Trust | Yes | Yes |
| RBD | Dorset County Hospital NHS Foundation Trust | Yes | Yes |
| RWH | East And North Hertfordshire NHS Trust | Yes | Yes |
| RJN | East Cheshire NHS Trust | Yes | Yes |
| RVV | East Kent Hospitals University NHS Foundation Trust | Yes | Yes |
| RXR | East Lancashire Hospitals NHS Trust | Yes | Yes |
| RDE | East Suffolk And North Essex NHS Foundation Trust | Yes | Yes |
| RXC | East Sussex Healthcare NHS Trust | Yes | Yes |
| RVR | Epsom And St Helier University Hospitals NHS Trust | Yes | Yes |
| RDU | Frimley Health NHS Foundation Trust | Yes | Yes |
| RR7 | Gateshead Health NHS Foundation Trust | Yes | Yes |
| RLT | George Eliot Hospital NHS Trust | Yes | Yes |
| RTE | Gloucestershire Hospitals NHS Foundation Trust | Yes | Yes |
| RN3 | Great Western Hospitals NHS Foundation Trust | Yes | Yes |
| RJ1 | Guy's And St Thomas' NHS Foundation Trust | Yes | Yes |
| RN5 | Hampshire Hospitals NHS Foundation Trust | Yes | Yes |
| RCD | Harrogate And District NHS Foundation Trust | Yes | Yes |
| RQX | Homerton Healthcare NHS Foundation Trust | Yes | No |
| RWA | Hull University Teaching Hospitals NHS Trust | Yes | Yes |
| RYJ | Imperial College Healthcare NHS Trust | Yes | Yes |
| R1F | Isle Of Wight NHS Trust | Yes | Yes |
| RGP | James Paget University Hospitals NHS Foundation Trust | Yes | Yes |
| RNQ | Kettering General Hospital NHS Foundation Trust | Yes | Yes |
| RJZ | King's College Hospital NHS Foundation Trust | Yes | Yes |
| RAX | Kingston And Richmond NHS Foundation Trust | Yes | Yes |
| RXN | Lancashire Teaching Hospitals NHS Foundation Trust | Yes | Yes |
| RR8 | Leeds Teaching Hospitals NHS Trust | Yes | Yes |
| RJ2 | Lewisham And Greenwich NHS Trust | Yes | Yes |
| REM | Liverpool University Hospitals NHS Foundation Trust | Yes | Yes |
| R1K | London North West University Healthcare NHS Trust | Yes | Yes |
| RWF | Maidstone And Tunbridge Wells NHS Trust | Yes | Yes |
| R0A | Manchester University NHS Foundation Trust | Yes | Yes |
| RPA | Medway NHS Foundation Trust | Yes | Yes |
| RBN | Mersey And West Lancashire Teaching Hospitals NHS Trust | Yes | Yes |
| RAJ | Mid And South Essex NHS Foundation Trust | Yes | Yes |
| RBT | Mid Cheshire Hospitals NHS Foundation Trust | Yes | Yes |
| RXF | Mid Yorkshire Teaching NHS Trust | Yes | Yes |
| RD8 | Milton Keynes University Hospital NHS Foundation Trust | Yes | Yes |
| RM1 | Norfolk And Norwich University Hospitals NHS Foundation Trust | Yes | Yes |
| RVJ | North Bristol NHS Trust | Yes | Yes |
| RNN | North Cumbria Integrated Care NHS Foundation Trust | Yes | Yes |
| RVW | North Tees And Hartlepool NHS Foundation Trust | Yes | Yes |
| RGN | North West Anglia NHS Foundation Trust | Yes | Yes |
| RNS | Northampton General Hospital NHS Trust | Yes | Yes |
| RM3 | Northern Care Alliance NHS Foundation Trust | Yes | Yes |
| RJL | Northern Lincolnshire And Goole NHS Foundation Trust | Yes | Yes |
| RTF | Northumbria Healthcare NHS Foundation Trust | Yes | Yes |
| RX1 | Nottingham University Hospitals NHS Trust | Yes | Yes |
| RTH | Oxford University Hospitals NHS Foundation Trust | Yes | Yes |
| RHU | Portsmouth Hospitals University NHS Trust | Yes | Yes |
| RHW | Royal Berkshire NHS Foundation Trust | Yes | Yes |
| REF | Royal Cornwall Hospitals NHS Trust | Yes | Yes |
| RH8 | Royal Devon University Healthcare NHS Foundation Trust | Yes | Yes |
| RAL | Royal Free London NHS Foundation Trust | Yes | Yes |
| RA2 | Royal Surrey County Hospital NHS Foundation Trust | Yes | Yes |
| RD1 | Royal United Hospitals Bath NHS Foundation Trust | Yes | Yes |
| RNZ | Salisbury NHS Foundation Trust | Yes | Yes |
| RXK | Sandwell And West Birmingham Hospitals NHS Trust | Yes | Yes |
| RCU | Sheffield Children's NHS Foundation Trust | Yes (Paediatric   - not included) | No |
| RHQ | Sheffield Teaching Hospitals NHS Foundation Trust | Yes | Yes |
| RK5 | Sherwood Forest Hospitals NHS Foundation Trust | Yes | Yes |
| RH5 | Somerset NHS Foundation Trust | Yes | Yes |
| RTR | South Tees Hospitals NHS Foundation Trust | Yes | Yes |
| R0B | South Tyneside And Sunderland NHS Foundation Trust | Yes | Yes |
| RJC | South Warwickshire University NHS Foundation Trust | Yes | Yes |
| RJ7 | St George's University Hospitals NHS Foundation Trust | Yes | Yes |
| RWJ | Stockport NHS Foundation Trust | Yes | Yes |
| RTP | Surrey And Sussex Healthcare NHS Trust | Yes | Yes |
| RMP | Tameside And Glossop Integrated Care NHS Foundation Trust | Yes | Yes |
| RNA | The Dudley Group NHS Foundation Trust | Yes | Yes |
| RAS | The Hillingdon Hospitals NHS Foundation Trust | Yes | Yes |
| RTD | The Newcastle Upon Tyne Hospitals NHS Foundation Trust | Yes | Yes |
| RQW | The Princess Alexandra Hospital NHS Trust | Yes | Yes |
| RCX | The Queen Elizabeth Hospital, King's Lynn, NHS Foundation Trust | Yes | Yes |
| RFR | The Rotherham NHS Foundation Trust | Yes | Yes |
| RL4 | The Royal Wolverhampton NHS Trust | Yes | Yes |
| RXW | The Shrewsbury And Telford Hospital NHS Trust | Yes | Yes |
| RA9 | Torbay And South Devon NHS Foundation Trust | Yes | Yes |
| RWD | United Lincolnshire Teaching Hospitals NHS Trust | Yes | Yes |
| RRV | University College London Hospitals NHS Foundation Trust | Yes | Yes |
| RHM | University Hospital Southampton NHS Foundation Trust | Yes | Yes |
| RRK | University Hospitals Birmingham NHS Foundation Trust | Yes | Yes |
| RA7 | University Hospitals Bristol And Weston NHS Foundation Trust | Yes | Yes |
| RKB | University Hospitals Coventry And Warwickshire NHS Trust | Yes | Yes |
| R0D | University Hospitals Dorset NHS Foundation Trust | Yes | Yes |
| RTG | University Hospitals Of Derby And Burton NHS Foundation Trust | Yes | Yes |
| RWE | University Hospitals Of Leicester NHS Trust | Yes | Yes |
| RTX | University Hospitals Of Morecambe Bay NHS Foundation Trust | Yes | Yes |
| RJE | University Hospitals Of North Midlands NHS Trust | Yes | Yes |
| RK9 | University Hospitals Plymouth NHS Trust | Yes | Yes |
| RYR | University Hospitals Sussex NHS Foundation Trust | Yes | Yes |
| RBK | Walsall Healthcare NHS Trust | Yes | Yes |
| RWW | Warrington And Halton Teaching Hospitals NHS Foundation Trust | Yes | Yes |
| RWG | West Hertfordshire Teaching Hospitals NHS Trust | Yes | Yes |
| RGR | West Suffolk NHS Foundation Trust | Yes | Yes |
| RKE | Whittington Health NHS Trust | Yes | Yes |
| RBL | Wirral University Teaching Hospital NHS Foundation Trust | Yes | Yes |
| RWP | Worcestershire Acute Hospitals NHS Trust | Yes | Yes |
| RRF | Wrightington, Wigan And Leigh NHS Foundation Trust | Yes | Yes |
| RLQ | Wye Valley NHS Trust | Yes | Yes |
| RCB | York And Scarborough Teaching Hospitals NHS Foundation Trust | Yes | Yes |
| RVN | Avon And Wiltshire Mental Health Partnership NHS Trust | No | No |
| RWX | Berkshire Healthcare NHS Foundation Trust | No | No |
| RXT | Birmingham And Solihull Mental Health NHS Foundation Trust | No | No |
| RYW | Birmingham Community Healthcare NHS Foundation Trust | No | No |
| TAJ | Black Country Healthcare NHS Foundation Trust | No | No |
| TAD | Bradford District Care NHS Foundation Trust | No | No |
| RY2 | Bridgewater Community Healthcare NHS Foundation Trust | No | No |
| RT1 | Cambridgeshire And Peterborough NHS Foundation Trust | No | No |
| RYV | Cambridgeshire Community Services NHS Trust | No | No |
| RV3 | Central And North West London NHS Foundation Trust | No | No |
| RYX | Central London Community Healthcare NHS Trust | No | No |
| RXA | Cheshire And Wirral Partnership NHS Foundation Trust | No | No |
| RJ8 | Cornwall Partnership NHS Foundation Trust | No | No |
| RYG | Coventry And Warwickshire Partnership NHS Trust | No | No |
| RX4 | Cumbria, Northumberland, Tyne And Wear NHS Foundation Trust | No | No |
| RY8 | Derbyshire Community Health Services NHS Foundation Trust | No | No |
| RXM | Derbyshire Healthcare NHS Foundation Trust | No | No |
| RWV | Devon Partnership NHS Trust | No | No |
| RDY | Dorset Healthcare University NHS Foundation Trust | No | No |
| RYK | Dudley Integrated Health And Care NHS Trust | No | No |
| RWK | East London NHS Foundation Trust | No | No |
| RX9 | East Midlands Ambulance Service NHS Trust | No | No |
| RYC | East Of England Ambulance Service NHS Trust | No | No |
| R1L | Essex Partnership University NHS Foundation Trust | No | No |
| RTQ | Gloucestershire Health And Care NHS Foundation Trust | No | No |
| RP4 | Great Ormond Street Hospital For Children NHS Foundation Trust | No (Paediatric   - not included) | No |
| RXV | Greater Manchester Mental Health NHS Foundation Trust | No | No |
| RW1 | Hampshire And Isle Of Wight Healthcare NHS Foundation Trust | No | No |
| R1A | Herefordshire And Worcestershire Health And Care NHS Trust | No | No |
| RY4 | Hertfordshire Community NHS Trust | No | No |
| RWR | Hertfordshire Partnership University NHS Foundation Trust | No | No |
| RV9 | Humber Teaching NHS Foundation Trust | No | No |
| RXY | Kent And Medway NHS And Social Care Partnership Trust | No | No |
| RYY | Kent Community Health NHS Foundation Trust | No | No |
| RW5 | Lancashire & South Cumbria NHS Foundation Trust | No | No |
| RGD | Leeds And York Partnership NHS Foundation Trust | No | No |
| RY6 | Leeds Community Healthcare NHS Trust | No | No |
| RT5 | Leicestershire Partnership NHS Trust | No | No |
| RY5 | Lincolnshire Community Health Services NHS Trust | No | No |
| RP7 | Lincolnshire Partnership NHS Foundation Trust | No | No |
| RBQ | Liverpool Heart And Chest Hospital NHS Foundation Trust | No | No |
| REP | Liverpool Women's NHS Foundation Trust | No (Women’s   - not included) | No |
| RRU | London Ambulance Service NHS Trust | No | No |
| RW4 | Mersey Care NHS Foundation Trust | No | No |
| RRE | Midlands Partnership University NHS Foundation Trust | No | No |
| RP6 | Moorfields Eye Hospital NHS Foundation Trust | No | No |
| RMY | Norfolk And Suffolk NHS Foundation Trust | No | No |
| RY3 | Norfolk Community Health And Care NHS Trust | No | No |
| RX6 | North East Ambulance Service NHS Foundation Trust | No | No |
| RAT | North East London NHS Foundation Trust | No | No |
| G6V2S | North London NHS Foundation Trust | No | No |
| RLY | North Staffordshire Combined Healthcare NHS Trust | No | No |
| RX7 | North West Ambulance Service NHS Trust | No | No |
| RP1 | Northamptonshire Healthcare NHS Foundation Trust | No | No |
| RHA | Nottinghamshire Healthcare NHS Foundation Trust | No | No |
| RNU | Oxford Health NHS Foundation Trust | No | No |
| RPG | Oxleas NHS Foundation Trust | No | No |
| RT2 | Pennine Care NHS Foundation Trust | No | No |
| R0C | Project Nightingale NHS Trust | No | No |
| RPC | Queen Victoria Hospital NHS Foundation Trust | No | No |
| RXE | Rotherham Doncaster And South Humber NHS Foundation Trust | No | No |
| RAN | Royal National Orthopaedic Hospital NHS Trust | No | No |
| RGM | Royal Papworth Hospital NHS Foundation Trust | No | No |
| TAH | Sheffield Health & Social Care NHS Foundation Trust | No | No |
| R1D | Shropshire Community Health NHS Trust | No | No |
| RYE | South Central Ambulance Service NHS Foundation Trust | No | No |
| RYD | South East Coast Ambulance Service NHS Foundation Trust | No | No |
| RV5 | South London And Maudsley NHS Foundation Trust | No | No |
| RYQ | South London Healthcare NHS Trust | No | No |
| RQY | South West London And St George's Mental Health NHS Trust | No | No |
| RXG | South West Yorkshire Partnership NHS Foundation Trust | No | No |
| RYF | South Western Ambulance Service NHS Foundation Trust | No | No |
| RXX | Surrey And Borders Partnership NHS Foundation Trust | No | No |
| RDR | Sussex Community NHS Foundation Trust | No | No |
| RX2 | Sussex Partnership NHS Foundation Trust | No | No |
| RNK | Tavistock And Portman NHS Foundation Trust | No | No |
| RX3 | Tees, Esk And Wear Valleys NHS Foundation Trust | No | No |
| RBV | The Christie NHS Foundation Trust | No | Yes |
| REN | The Clatterbridge Cancer Centre NHS Foundation Trust | No | No |
| RL1 | The Robert Jones And Agnes Hunt Orthopaedic Hospital NHS Foundation Trust | No | No |
| RPY | The Royal Marsden NHS Foundation Trust | No | No |
| RRJ | The Royal Orthopaedic Hospital NHS Foundation Trust | No | No |
| RET | The Walton Centre NHS Foundation Trust | No | Yes |
| RKL | West London NHS Trust | No | No |
| RYA | West Midlands Ambulance Service University NHS Foundation Trust | No | No |
| RY7 | Wirral Community Health And Care NHS Foundation Trust | No | No |
| RX8 | Yorkshire Ambulance Service NHS Trust | No | No |
| Abbreviations: AA, andexanet alfa; NHS, national health service; ODS, organisation data service  *Trusts with 24 hour consultant-led emergency care activity reported in the last six months.[(](https://paperpile.com/c/SMs6PJ/S3rK)10) | | | |
